# Abstract of the Proceedings of the Buffalo Medical Association

**Published:** 1862-01

**Authors:** J. F. Miner

**Affiliations:** Secretary


ART II.—*Abstract af the Proceedings of the Buffalo Medical Association.*

TUESDAY EVENING, Dec. 4, 1861.

The Association met at the usual hour.

Present—Dr. C. C. F. Gay, President, in the Chair; Drs. White, Samo, Gould, Miner, Congar, Whitney, Kempson.

*Dr. E. P. Gray's* application for membership elicited considerable discussion, in which most of the members present participated.

*Dr. Whitney* represented that Dr. Gray desired to become a member of the Association, and to mingle more in the brotherhood of physicians. Spoke at length of the action of the Erie County Medical Society, in relation to Drs. Hill and Gray, and says he knows Dr. Gray most heartily regrets his action in that matter. Could say much more representing truly Dr. Gray's position and sentiments, but has not been authorized to do so. Knows that this application is made in good faith, and from pure motive.

*Dr. E. P. Gray* was unanimously elected a member upon compliance with the By-Laws.

Voted that the Committee to audit Treasurer's account be granted more time.

*Dr. Gould* presented a small fibrous tumor, which he had removed from the second joint of the thumb, of an infant immediately after birth. It was the size of a grape, and attached by a slim pedicle about the size of a grape stem.

*Dr. Whitney* was reminded by Dr. Gould's specimen of a case, where he found tumor over the upper incisor tooth, in a boy 14 years old. It had been twice excised by the family physician, but soon returned, and was sent to him for treatment. Ligated it and it soon dropt off; seemed inclined to grow, and caustic was applied; at last forced an instrument down upon the alveorla process detaching it thoroughly from the bone, and after this operation it gave no further trouble, never afterwards returned.

*Dr. Gould* reported the following case of abscess of the liver, occurring with phthisis:—Mrs. H. aged 26 years, had been in delicate health for twelve years; married about six years; never pregnant; first saw her Feb. 6th, 1860, and attended her for several months; had a bad cough and severe pain in the abdomen and hip; finally recovered her usual health, which was at no time very good.

*March 6th*, 1861.—He was called to attend her again; her cough was constant and she complained of severe pain in the right hypochondriac region, which continued in defiance of all remedies; after a few days a small tumor was discovered to the right and a little above the umbilicus, which gradually increased in size; tuberculosis was easily diagnosed, but the character of the tumor was not so easily decided upon. Dr. Miner examined the case in consultation, and subsequently Dr. Rochester also examined it and diagnosed the case, as it proved to be abscess of the liver; the patient continued to sink, and died on the 27th of April. On making a post-mortem examination, Drs. Rochester, Miner, Gay, White and Wilcox, being present, an enormous abscess of the liver was found occupying nearly the whole right side of the abdomen, forcing itself far down in the inguinal region, and containing a quart or more of pus. The lungs were nearly destroyed by tuberculosis.

*Prof. White* related a case of somewhat similar disease occurring in this city, and the patient still residing here. The abscess opened externally, and biliary calculi were discharged; also, mentioned the case of a Mr. Trumbull, who as early as 1832, had intermittent fever, which was arrested by quinine, but he still remained quite ill; finally had pain in right side, tenderness, fever, severe cough, and at length expectorated bile, demonstrating that the hepatic abscess had discharged through the lungs. These cases are interesting, as showing the remarkable powers of nature, since this patient has nearly or quite regained his usual health and strength.—They are also interesting from their comparative rarity, and the great difficulty of early and satisfactory diagnosis. Spoke of the different directions in which hepatic abscess may discharge itself, and the various results attending it; remarking that, as is well known, a cure of hepatic abscess may be effected after the discharge of its contents in any of the various methods, or it may result without this occurrence, from more or less complete absorption of the pus by the membrane investing the sides of the abscess. This is shown upon post-mortem examina tion, when the walls of the abscess are found to have united, forming a callous cicatrix, and not unfrequently a remnant of pus, which has been converted into a chasy concretion, may still be found locked up in the tissue forming the cicatrix.

*Dr. Gay* reported in brief, a case of rupture of the perineum, upon which he had recently operated, with what success could not yet fully determine, since but two weeks had elapsed and the union was not yet complete

The rupture involved the sphincter muscles of the anus, and some portion of the recto-vaginal septum; was caused by labor some two years since, while attended by a very good physician.

After preparing the parts by thorough freshening the edges, two silver sutures were applied through the recto-vaginal septum, and three larger ones through the perineum. The second day after the operation there were several dejections from the bowels, which seemed to have passed naturally, and not to have produced any trouble or disturbance with the parts operated upon.

There were several points of interest in the case, and at a later period when the results of the operation could be stated fully, would report more in detail.

*Dr. Miner* spoke of a case occurring in the Buffalo General Hospital, under the care of the attending physician; rupture extended down to the virge of the anus, but not entirely through the sphincter muscles.—Three months later, she was admitted to the surgical ward, and operated upon in the usual manner, with quilled suture. Second day after operation, menstruation commenced, though that process had but just been completed, and on the fifth day she was attacked with most violent diarrhœa, which continued for thirty-six hours, with great severity, and could not be wholly controlled for three or four days. Notwithstanding these most adverse accidents the union was complete.

*Dr. M.* spoke also of another case upon which he had operated with most complete success, though menstruation commenced soon after the operation. Thought the excitement attending the operation was the cause of the menstrual secretion being so often produced at the unnatural season, and regarded it as more likely to prevent union than diarrhœa.

Judging from his personal experience and observation, would conclude that the dangers of non-union after operation for rupture of the perineum, had been greatly overrated; was confirmed in this opinion by often observing pretty severe rupture unite without any effort other than position, cleanliness and rest, the bowels, being controlled by anodynes. If union will thus generally take place in recent cases, while yet the lochial discharge is present; could see no good reason to conclude that any great uncertainty could exist after operation. When the edges are pared, and well approximated, it seems almost certain that it will be followed by satisfactory results

*Dr. Gould* related a case of very severe rupture and laceration of vagina, attended by a midwife. Portions of the vagina hung out externally and were removed; the result was a perfect union of the vagina, the walls adhering fully and perfectly; did not know what was the final result; thought that menstruation never returned since the patient was quite advanced in age.

*Dr. Kempson* is astonished that Dr. Miner should intimate former doubts, and now increased confidence in the success of this operation.—Has formerly attended a very large number of obstetrical cases, and regards it singular that he has never met but six cases of rupture of the perineum, two of these were ruptured very extensively, and all recovered without operation; never saw the operation only in this country; thinks it more common here; that the tissues stretch more in England than in this country; is very much astonished at its great frequency in America.

*Dr. Miner* explained his meaning in reference to spontaneous union, not designing to intimate that a case like the one related by Dr. Gay, would unite while recent by proper rest, cleanliness, &c., &c., but that simple laceration would often do so, and extensive laceration would do so no doubt, if properly approximated by suitable suture.

*Dr. Kempson* related the case of a very respectable lady who had perineal laceration; was operated upon in Toronto, with almost perfect success, a small opening only, remaining in the recto vaginal septum; soon after, she found herself pregnant, and was filled with horror at the idea of a second labor, and possible laceration. Several physicians were consulted, and the opinion prevailed that she could not be delivered at full term without producing rupture of the old cicatrix, and that it was not best to risk a second operation, sinee now she was very comfortable. Premature labor, or abortion, was induced at about the fourth month, and terminated very successfully. Dr. K. desired to know when, (if at all,) such operation should be made.

*Dr. Gay* expressed the opinion that the fourth month was a very proper time for such operation.

*Dr. Miner* gave reasons why a physician should decline the operation under the circumstances as related by Dr. K. though he had no doubt that her physicians were in possession of facts, which would in this instance justify the operation, and would not judge of this case, or speak with particular reference to it, though the lady's great anxiety and unwillingness to be delayed was ground for suspicion that she had other motives than were expressed. Great danger, or even certainty, of severe perineal laceration, could not be unhesitatingly regarded as justifying the operation for abortion, though it might,and would,be ample justification for inducing premature labor at such time as would afford reasonáble expectation of saving the life of the child, provided this was regarded as affording sufficient security to the mother.

*Dr. Congar* was satisfied that it would not answer for him to produce abortion to prevent any laceration, however severe. Thought that no good authority would be found to favor such practice. Dr. Miner had remarked truly upon the impropriety of such operation from such motive. Sacrificing the life of the child from the fear of such injury to the mother, could not be regarded otherwise than improper. Persons of the greatest respectability might desire and insist upon such procedure, but for himself he could not but regard it as wrong; whatever influence was brought to bear, or however many consulting physicians might advise or favor such course, he could not adopt it.

*Dr. Kempson* explained more fully, and said that in the case he had related, the physicians in charge regarded it as not only endangering laceration, but as a matter of life and death; should not think it proper unless it was regarded as imperiling life. Hoped no one would think that they had for a moment forgotten that the life of the child was the life of a human being.

*Dr. Gould* remarked upon the difficulty of one physician judging for another in such matters. Spoke of the great frequency of applications for operation, or medicines for the purposes of abortion, and the great earnestness with which they would urge the importance or necessity of measures being adopted for relief, and lastly, when other means had failed, how sometimes patients would get instruments and operate upon themselves—would use all sorts of instruments and take all sorts of medicine for such purposes. Physicians were only safe when they make their own examinations and judge for themselves quite independently of any representations which may be made to them.

Voted to adjourn. J. F. MINER, Secretary.

